# Effects of Estrogen, Nitric Oxide, and Dopamine on Behavioral Locomotor Activities in the Embryonic Zebrafish: A Pharmacological Study

**DOI:** 10.3390/toxics4040024

**Published:** 2016-09-26

**Authors:** Vania Murcia, Luke Johnson, Meredith Baldasare, Bridgette Pouliot, John McKelvey, Brandon Barbery, Julie Lozier, Wade E. Bell, James E. Turner

**Affiliations:** Department of Biology, Center for Molecular, Cellular, and Biological Chemistry, Virginia Military Institute, Lexington, VA 24450, USA; murciava17@mail.vmi.edu (V.M.); johnsonlk17@mail.vmi.edu (L.J.); baldasaremp16@mail.vmi.edu (M.B.); pouliotba16@mail.vmi.edu (B.P.); mckelveyjm16@mail.vmi.edu (J.M.); barberyb18@mail.vmi.edu (B.B.); lozierja@vmi.edu (J.L.); bellwe@vmi.edu (W.E.B.)

**Keywords:** nitric oxide, estrogen, motor dysfunction, dopamine, 6-hydroxydopamine neuronal toxicity, zebrafish

## Abstract

Nitric oxide (NO) has been shown to affect motor function. Specifically, NO has been shown to act through regulation of dopamine (DA) release, transporter function, and the elicitation of neuroprotection/neurodegeneration of neurons. Recently, zebrafish have been proposed to be a new model for the study of various types of motor dysfunctions, since neurotoxin damage to their nigrostriatal-like neurons exhibit motor anomalies similar to those of mammalian models and human patients. Results from this study demonstrate that when NO synthesis is inhibited in zebrafish, using a neuronal NO synthase inhibitor (nNOSI), a condition called ‘listless’ occurs, where the fish lack swimming abilities, are rigid, and have difficulty maintaining balance. Additionally, co-treatment with either NO or estrogen (E2), an upstream regulator of NO synthase, can rescue fish from the ‘listless’ phenotype caused by exposure to the neurotoxin 6-hydroxydopamine (6 OHDA). In turn, NO deprived zebrafish were rescued from the ‘listless’ phenotype when co-treated with L-DOPA, a precursor to DA. Interestingly, the longer fish are exposed to a 6 OHDA + nNOSI co-treatment, the slower the recovery after washout, compared to a single treatment of each. Most significantly, NO involvement in the motor homeostasis of the embryonic zebrafish was shown to be expressed through the NO-cGMP-dependent pathway, and response to nNOSI treatments is developmentally regulated. In conclusion, these results indicate that there is a link between E2, NO, and DA systems that regulate motor functions in the embryonic zebrafish.

## 1. Introduction

Damage of the human nigrostriatal projection to the striatum of the basal ganglia (BG) causes motor dysfunctions associated with Parkinson’s disease (PD) [1,2,3]. Next to Alzheimer’s disease, PD is the second most prevalent neurodegenerative condition found among humans There are seven million people affected by PD globally (one million in the US) and 103,000 deaths annually (2013 figures, up from 44,000 in 1990) [4]. In the US alone, there is a total direct and indirect cost burden due to PD of $23 billion yearly [5]. Several signaling molecules have been implicated in the neuromodulation/neuroprotection of DA neurons in the nigorstrital (BG) pathway associated with either animal model of 1-methyl-4-phenyl-1,2,3,6-tetrahydropyridine (MPTP) or 6-hydrozydopamine (6OHDA) neurotoxicity, which create PD-like symptoms, or from PD patient clinical data. One of these signaling molecules is nitric oxide (NO). NO, a gas released by the actions of the nitric oxide synthase (NOS) enzyme on l-argenine, acts as a signaling molecule with direct actions on existing metabolic pathways, as well as through genomic mechanisms [6,7]. At higher concentrations, NO can act as a free radical in some situations or binds to superoxide anion (O_2_**^−^**), causing pathophysiological effects that act to harm the body [8,9,10]. It is under these conditions that NO is thought to play a role in the genesis of PD [11]. On the other hand, NO at lower concentrations can act as a cellular protectant through prevention of apoptosis, excitotoxicity, neuronal depolarization, and regulation of the redox state in the mitochondria [12,13]. Indeed, several studies have indicated that NOS containing neurons within the striatum of the BG are resistant to neurodegeneration [14]. NO acting at the cellular level interacts with either its soluble guanylyl cyclase (sGC) receptor molecule to produce cyclic GMP (cGMP) or causes *S*-nitrosylation of cysteine residues [15,16]. In the BG, NO has been shown to affect DA release, influence transporter function, and elicit neuroprotection of DA neurons [17,18]. NO synthesis inhibitors (NOSI) have also been shown to decrease spontaneous motor activity in various animal models, as well as the initiation of catalepsy, which is characterized by suspension of sensation, muscle rigidity, and fixity of posture [18]. The addition of DA D1 and D2 receptor agonists has been shown to decrease these NOSI-mediated motor deficits [18].

Estrogen (E2) is another signaling/hormonal molecule linked with numerous nervous system functions, including PD. Specifically, it has been well established through many in vivo and in vitro studies that E2 is critical to normal development, maintenance, neuroprotection, and function of the central nervous system [19]. A key observation linking E2 to PD is that there is a sexual dimorphism apparent in the human population based on various clinical studies. Specifically, women generally have a lower risk for PD than men. This changes after menopause, and, therefore, E2 is implicated to be a PD protectant in the female population [20]. Part of the mechanism by which E2 may exert its influence in the BG of PD patients is through its documented influence on the expression of NOS [21]. Therefore, more research is necessary to understand the effects of the E2-NO linkage and its actions on the intact and lesioned mature and developing nervous system, particularly in the BG, to better understand their possible role in PD.

The continual effort to development of a quick turnaround animal model that links E2-NO to both DA actions and motor functions is crucial for further understanding of how this interplay affects the stabilization of PD-like symptoms. In turn, a model where BG-like pathways are simpler and the DA neurons fewer in number and easier to visualize and access would be ideal for such studies. Embryonic zebrafish would appear to fit these criteria. The DA system has been well characterized in both embryo development and in adults [22,23]. Additionally, zebrafish embryos and adults respond to the DA neurotoxins MPTP and 6OHDA, as well as to the DA receptor agonists/antagonists, in much the same manner as mammalian models of PD [23,24,25]. Indeed, there are an increasing number of studies that make a case for the use of zebrafish as a model for the study of movement disorders, such as PD [25]. Recent observations from our lab have established a zebrafish locomotor dysfunction model, linked to both E2 and NO deficiency. Specifically, locomotor deficient zebrafish embryos were named ‘listless’ in response to E2 deprivation caused by aromatase inhibitor (AI, inhibits the enzyme responsible for E2 synthesis) treatment [26]. Our most recent results implied that NO may be the downstream mediator of an E2 rescue of fish from the ‘listless’ condition, but it also demonstrated that, in the absence of endogenous NO, fish also became listless [27,28]. Therefore, initial evidence for NO’s role in locomotor activities in an embryonic zebrafish model was established.

The results in the current study present both behavioral and pharmacological evidence that there is a link between E2, NO and DA systems that regulate motor functions in the embryonic zebrafish. Furthermore, these results substantiate earlier findings that the zebrafish will provide an additional model for the development of future therapeutic strategies in conditions of motor dysfunction, such as PD [25].

## 2. Materials and Methods

### 2.1. Fish Preparation

The compound *roy*; *nacre* double homozygous mutant zebrafish, which is named *casper*, was used in this study. *Casper* shows the effect of combined melanocyte and iridophore loss, in which the bodies of embryonic and adult fish are largely transparent due to loss of light absorption and reflection [29]. Upon arrival, fish were placed in an autoclaved embryo rearing salt solution (ERS) composed of 0.04 g of CaCl_2_, 0.163 g of MgSO_4_, 1.0 g of NaCl, and 0.03 g of KCl, all in deionized water containing a 0.05% methylene blue solution, which serves as an antimicrobial agent. All solutions were changed every 24 h, and embryos were incubated at 28 °C. All reagents were obtained from Sigma-Aldrich unless noted otherwise, and solutions were made daily before use. A protease concentration of 2 mg/mL (Sigma, Saint Louis, MO, USA) was used to dechorionate embryos treated at 48 h post fertilization (hpf), in order to better expose the fish to the various treatments [28]. Fish treated at 4–6 days post fertilization (dpf) were allowed to hatch on their own. All procedures were in accordance with NIH guidelines for the care and treatment of animals. All data reported were derived from at least triplicate repeats of 20–30 fish per group.

### 2.2. Reagent Preparations

#### 2.2.1. NO/E2 Related Reagents

All NO-related reagents for treating zebrafish were tested in a dose response paradigm to ensure optimal results and proper survival. Based on the literature, baseline target concentrations were identified. Nitro-l-arginine methyl ester hydrochloride (l-NAME-hydrochloride, Sigma) was used as a general NOS inhibitor (gNOSI) at concentrations of 10, 15, 25, and 30 mM, prepared in ERS. gNOSI inhibits all three isoforms of NOS by acting as an l-arginine analog [30]. The optimal concentration for l-NAME solution was found to be 15 mM [31].

Proadifen hydrochloride (Sigma) was used as a selective nNOS inhibitor (nNOSI). With ERS as the diluent, fish were tested at 10 μM, 30 μM, and 50 μM. The 50-μM concentration provided optimal results in creating the listless condition.

1*H*-[1,2,4]Oxadiazolo[4,3-*a*]quinoxalin-1-one (ODQ, Sigma) was used as a soluble guanylyl cyclase (sGC) inhibitor, which compromises the NO-cGMP-dependent pathway by reducing cGMP production. It was dissolved into a 0.1% DMSO solution then diluted with ERS to a working concentration of 30 μM for application. In addition, DTT (dithiothreitol, Sigma) was used as an inhibitor of the NO-cGMP-independent pathway, which prevents *S*-nitrosylation events at a concentration of 100 µM.

Diethylenetriamine/nitric oxide adduct (DETA-NO, Sigma) was used to provide a slow extended release of exogenous NO as a co-treatment with some of the inhibitors used in the experiments in an effort to show that NO inhibition mediated symptoms exhibited by fish can be rescued. It was dissolved into ERS, resulting in working concentrations of 400 μM to 50 μM with 50 μM providing the best results.

E2 (17β-Estradiol, Sigma) was used at 10^−8^ M, as established previously [26] and initially solubilized in a 100% ethanol stock solution diluted down to the base treatment solution with ERS, ensuring that the ethanol concentration in the final solution was equal to or lower than 0.5%. The control group consisted of ERS salt solution, which contained 0.05% ethanol.

#### 2.2.2. DA Related Reagents

Various pharmacological agents were used to manipulate the zebrafish DA neurons. The neurotoxin 6-hydroxy-dopamine (6-OHDA, Sigma) was used at a concentration of 250–500 µM to illicit motor deficits by damaging DA neurons in the equivalent of the zebrafish nigrostriatal-like pathway, as established previously in the literature [24]. The l-DOPA DA precursor l-dopa ethyl ester (Sigma) was used at concentrations up to 10 mM, which was the limit of its solubility in the ERS control solution, and was used to elevate the neurotransmitter in deficient fish, starting at ranges prescribed previously for zebrafish embryos [32,33].

### 2.3. Research Protocols

#### 2.3.1. NO Response Systems in Embryonic Zebrafish

Two types of NOSI compounds, l-NAME, which interacts with all three NOS isoforms and nNOSI, a specific inhibitor of the nNOS isoform, were tested for their efficacy in creating the listless/PD–like phenotype. Specifically, fish were treated for 24 and 48 hpt and assessed for the phenotype. nNOSI was found to be the most desirable of the two, and was subsequently used throughout the remainder of the study. Next, fish were treated at 2, 4, and 6 days post fertilization (dpf) to determine which developmental age was most responsive to nNOSI-induced sensory-motor deficits, as well as recovery of function after washout with ERS. Specifically, at 24 and 48 h post treatment (hpt), fish were analyzed for spontaneous movement, equilibrium/balance ability, and probe-induced swimming movements. At 48 hpt, nNOSI treated fish were rinsed several times and changed out into ERS control solution, and followed at 8, 16 and 24 h post-washout (hpw) to determine the extent of recovery of sensory-motor functions.

#### 2.3.2. NO-sGC-cGMP-Dependent/Independent Pathway Analyses

Fish were treated at 6 dpf with either ODQ (Sigma, the NO-sGC-dependent pathway inhibitor) or DTT (Sigma, the NO-sGC-independent pathway inhibitor) to determine which inhibitor mimicked the typical sensory-motor deficits seen in the nNOSI treatments. Specifically, groups consisted of ERS controls, nNOSI, ODQ, and DTT treatments. At 48 h post treatment (hpt), fish were analyzed for spontaneous movement, equilibrium/balance ability, and probe-induced swimming movements.

#### 2.3.3. NO/E2 Rescue and Recovery from 6OHDA and nNOSI-Induced Listless/PD-Like Symptoms

To determine if NO could rescue fish from 6-OHDA toxicity, groups were treated at 6 dpf with ERS, 6OHDA, DETA-NO, or a 6OHDA + DETA-NO co-treatment. To determine if E2 depends on its downstream NOS target to rescue fish from 6OHDA toxicity, groups were treated for 24 h in either ERS control solution, E2, 6-OHDA, 6-OHDA + E2, and 6-OHDA + E2 + nNOSI. At 24 hpt fish were analyzed for spontaneous movement, equilibrium/balance ability, and probe-induced swimming movements. Additional experiments were designed to investigate the ability of nNOSI to rescue fish from 6-OHDA neurotoxicity. Specifically, fish were treated with nNOSI, 6-OHDA, or co-treatment with both (6-OHDA + nNOSI), and analyzed for survival over 72 h. In addition, to determine if l-DOPA can play a role in the rescue of fish from the nNOSI-induced listless/PD-like phenotype, groups were treated at 6 dpf with ERS, nNOSI, l-DOPA, or a l-DOPA + nNOSI co-treatment. At 48 hpt, fish were analyzed for spontaneous movement, equilibrium/balance ability, and probe-induced swimming movements.

### 2.4. Sensory-Motor Function Assays

The listless model was established through a series of earlier studies as a loss of sensory-motor function due to either E2 or NO depravation [26,27,28]. Specifically, this loss of sensory-motor function was defined by the exhibition of three neurological characteristics: The inability of fish to right themselves (vestibular dysfunction), the exhibition of no spontaneous swimming movements, and the inability to exhibit any movement when probed. The experimental fish were compared to conditions of the control fish in ERS. The ‘listless’ phenotype was observed under a dissecting microscope and was calculated as a percent of the treated group.

### 2.5. Data Analysis

Thirty zebrafish embryos were used in each experimental and control group within the various experimental paradigms described above. Each experimental study was repeated a minimum of three times for statistical interpretation. Data were analyzed for significant differences, either by a *z*-test for two population proportions or using a chi-square contingency table test for multiple proportions, followed by a Marascuilo’s post hoc analysis.

## 3. Results

### 3.1. There Is a Developmental Regulation of the NO Response System in Embryonic Zebrafish

**Figure 1:**
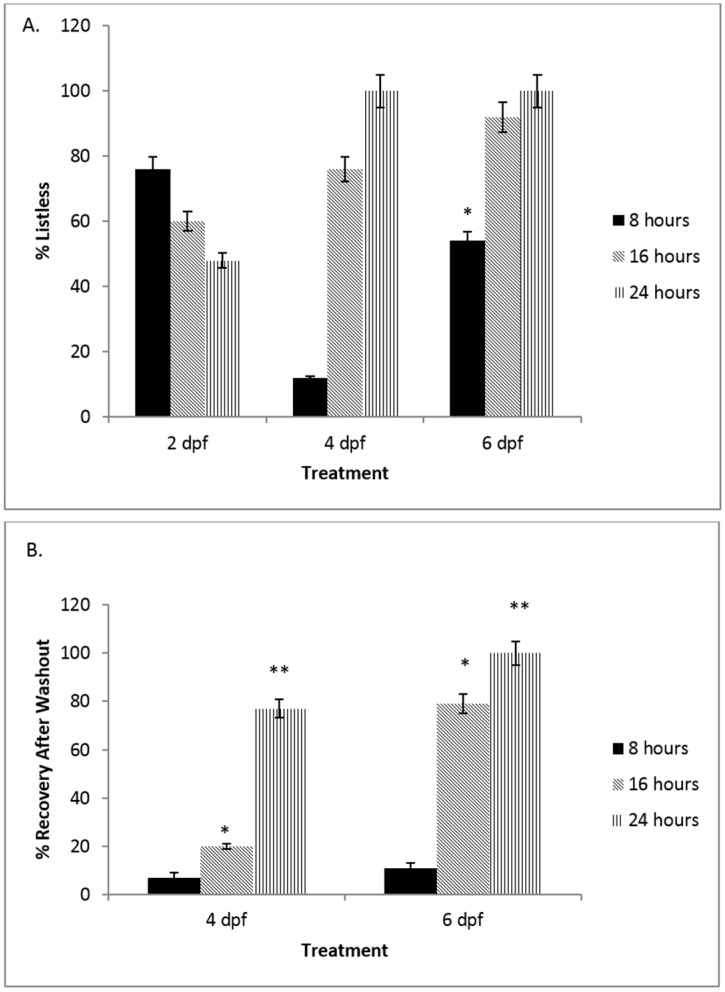
Analysis of the most desirable developmental age to study NO deprivation in the 2–6 days post fertilization (dpf) zebrafish. (**A**) The effect of developmental age on embryonic zebrafish response to 50 µM NO synthase inhibitor (NO synthase inhibitor (nNOSI) in developing the ‘listless’ phenotype. After 24 h of treatment, only 50% of fish at 2 dpf express the phenotype compared to 100% of both the 4 and 6 dpf groups (* *p* < 0.05). Additionally, note that there is no significant difference in the generation of the phenotype between the 4 and 6 dpf groups (* *p* > 0.05), except for the early phenotype acceleration at 8 h post treatment in the 6 dpf fish (* *p* < 0.05). Furthermore, the 2 dpf response to nNOSI treatment is not robust and is significantly less (* *p* < 0.05) than that exhibited by 4 and 6 dpf fish; (**B**) fish can quickly recover from the listless symptoms caused by nNOSI treatment. Specifically, fish treated with nNOSI at 6 dpf for 24 h and washed out with ERS recover 100% of their sensory-motor functions compared to only 80% of 4 dpf treated fish (* *p* < 0.05). Bars equal ± standard deviation (SD).

Figure 1 shows the analysis of the most desirable developmental age to study NO deprivation in the 2–6 days post fertilization (dpf) zebrafish. Figure 1A demonstrates the effect of developmental age on embryonic zebrafish response to 50 µM nNOSI in initiating the ‘listless’ phenotype. After 24 h of treatment, only 50% of fish at 2 dpf express the phenotype compared to 100% of both the 4 and 6 dpf groups (*p* < 0.05). Additionally, note that there is no significant difference in the generation of the phenotype between the 4 and 6 dpf groups (* *p* > 0.05) except for the significant early phenotype acceleration at 8 h post treatment in the 6 dpf fish (*p* < 0.05). Furthermore, the 2 dpf response to nNOSI treatment is not robust and is significantly less (*p* < 0.05) than that exhibited by 4 and 6 dpf fish. In Figure 1B, it can be seen that fish quickly recover from the listless symptoms caused by nNOSI treatment. Specifically, fish treated with nNOSI at 6 dpf for 24 h and washed out with ERS recover 100% of their sensory-motor functions compared to only 80% of 4 dpf treated fish (*p* < 0.05).

### 3.2. NO and E2 Deficiencies Cause Severe Sensory-Motor Deficits in the Embryonic Zebrafish Which Act through the NO-sGC-cGMP-Dependent Pathway

**Figure 2:**
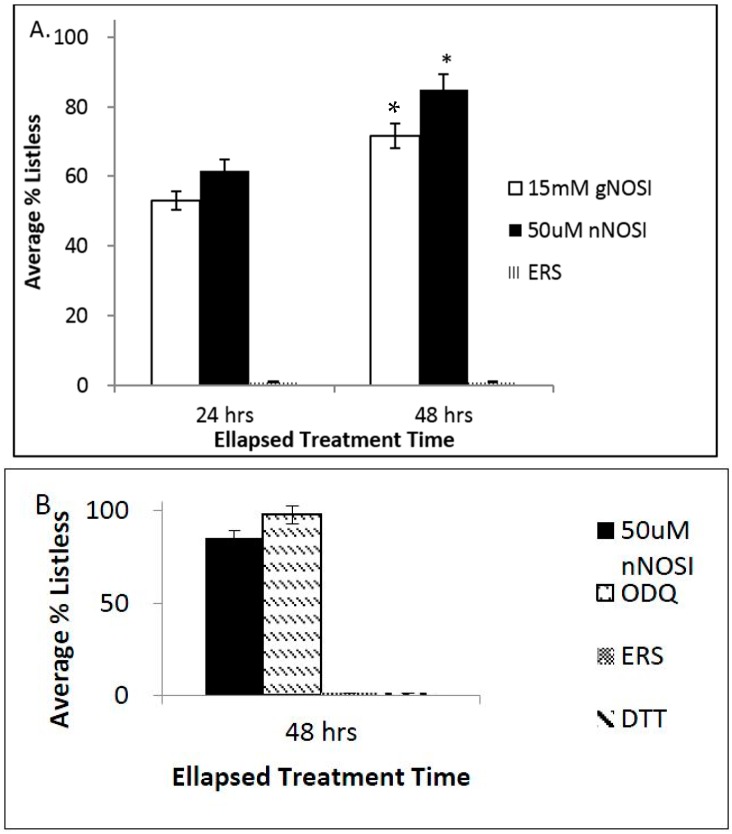
The effects of both general NOS inhibitor (gNOSI) and nNOSI on creating the listless/PD-like symptoms in 6 pdf treated zebrafish and the type of NO pathway engaged in this type of phenotype. (**A**) Both gNOSI and nNOSI create the listless symptoms over a 48 h treatment period; however, the nNOSI results are significantly greater (* *p* < 0.05) than that of gNOSI treatments at approximately 90% demonstrating the listless/PD-like symptoms compared to 75%, respectively; (**B**) Oxadiazolo[4,3-a]quinoxalin-1-one (ODQ) treatment, a sGC inhibitor in the NO/sGC/cGMP-dependent pathway, initiates the listless phenotype in a manner similar to that of nNOSI, where DTT, an *S*-nitrosilation NO/sGC/cGMP-independent pathway inhibitor, has no effect and is not significantly different form that of the ERS controls (* *p* > 0.05). Bars equal ± SD.

Figure 2 shows the effects of both gNOSI and nNOSI on creating the listless/ Parkinson’s disease (PD)-like symptoms in 6 pdf treated zebrafish and the type of NO pathway engaged in this type of phenotype. In Figure 2A both gNOSI and nNOSI are shown to create the listless symptoms over a 48 h treatment period; however, the nNOSI results are significantly greater (*p* < 0.05) than those of gNOSI treatments at approximately 90%, demonstrating the listless symptoms compared to 75%, respectively. Figure 2B indicates that ODQ treatment, a sGC inhibitor in the NO/sGC/cGMP-dependent pathway, initiates the listless/PD-like phenotype in a manner significantly different from that of nNOSI (*p* > 0.05), where DTT, an *S*-nitrosilation NO/sGC/cGMP-independent pathway inhibitor, has no effect and is not significantly different from that of the ERS controls (*p* > 0.05).

### 3.3. Both NO and E2 Can Rescue Fish from Severe Sensory-Motor Deficits Elicited by the Neurotoxin 6OHDA; However, E2 Rescue Is Dependent on the Downstream NO Pathway

**Figure 3:**
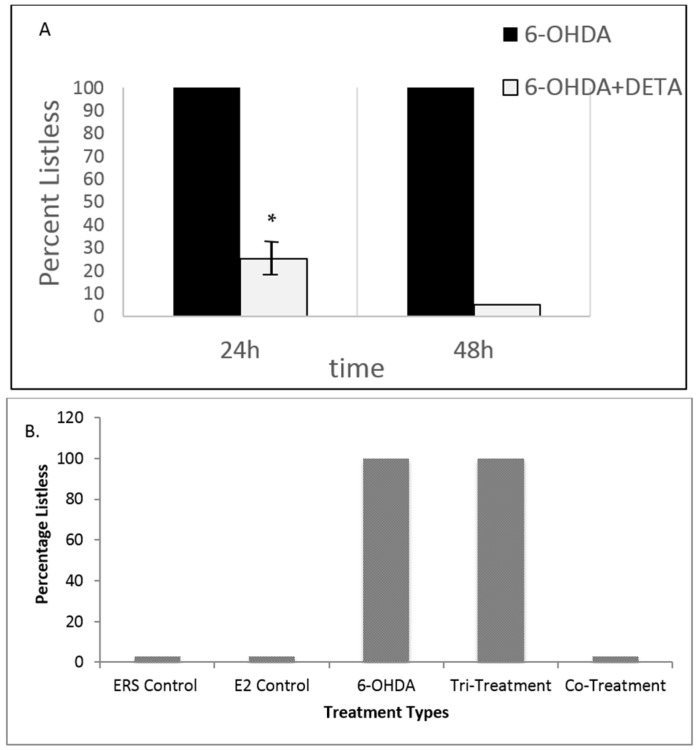
Rescue of 6 dpf fish from the 6OHDA-induced listless/PD-like phenotype under various treatment conditions. (**A**) DETA-NO co-treatment rescues fish from 6OHDA-induced listless symptoms. Specifically, at 24 and 48 h after treatment 100% of the 6OHDA treated population of fish exhibit listless/PD-like symptoms. In contrast, the co-treatment (6OHDA + DETA-NO) significantly (* *p* < 0.05) rescues fish from this phenotype at both 24 and 48 h after treatment. Specifically, by 24 h of co-treatment only approximately 25% of fish demonstrated the listless phenotype, while at 48 h all fish had recovered; (**B**) the effects of estrogen (E2) on 6OHDA treated fish. At 24 h after treatment, 100% of the 6OHDA treated population of fish exhibit listless/PD-like symptoms. In contrast, the co-treatment (6OHDA + E2) rescues 100% of fish from this phenotype at 24 h after treatment. However, the tri-treatment (6OHDA + E2 + nNOSI) eliminates all effects of the E2 co-treatment rescue phenomenon. In contrast, ERS and E2 treatments alone show no listless/PD-like phenotypes. Bars represent ± SD.

Figure 3 demonstrates the rescue of 6 dpf fish from the 6OHDA-induced listless/PD-like phenotype under various treatment conditions. Specifically, Figure 3A shows that DETA-NO co-treatment rescues fish from 6OHDA-induced listless symptoms. At 24 and 48 h after treatment, 100% of the 6OHDA treated population of fish exhibited listless-like symptoms. In contrast, the co-treatment (6OHDA + DETA-NO) significantly (*p* < 0.05) rescues fish from this phenotype at both 24 and 48 h after treatment. Specifically, by 24 h of co-treatment, only approximately 25% of fish demonstrated the listless/PD-like phenotype, and by 48 h all fish had recovered. In Figure 3B, the effects of E2, an upstream regulator of NOS, on 6OHDA treated fish are demonstrated. Specifically, at 24 h after treatment, 100% of the 6OHDA treated population of fish exhibited listless symptoms (*p* < 0.001). In contrast, the co-treatment (6OHDA + E2) rescues 100% of fish from this phenotype at 24 h after treatment. However, the tri-treatment (6OHDA + E2 + nNOSI) eliminates all effects of the E2 co-treatment rescue phenomenon (*p* < 0.001). In contrast, ERS and E2 treatments alone show no listless/PD-like phenotypes.

### 3.4. Embryonic Fish Treated with 6OHDA Recover at Rates Dependent on Exposure Time after Removal of the Neurotoxin and Survive Longer When Co-Treated with nNOSI

**Figure 4:**
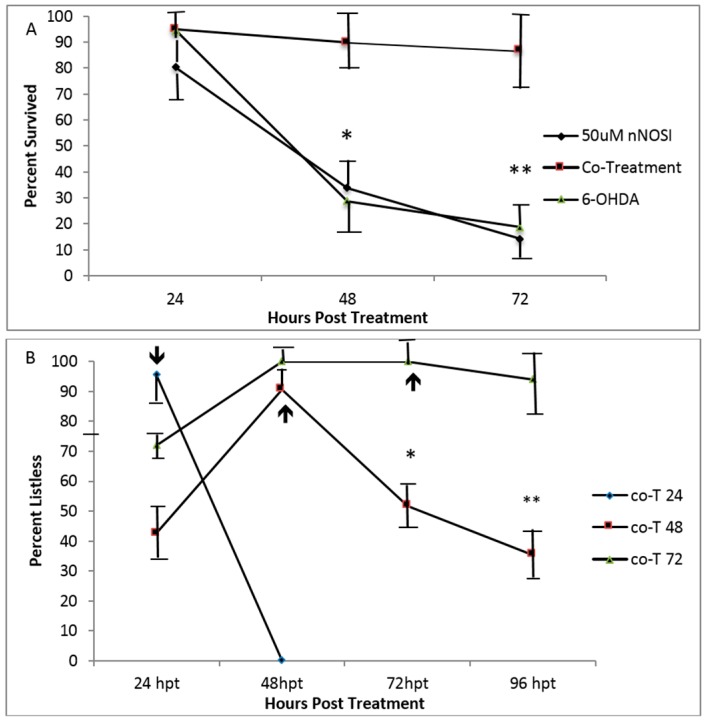
Analysis of the ability of 6 dpf treated fish to recover from 6 OHDA, nNOSI, and nNOSI + 6 OHDA co-treatments under various conditions. (**A**) Shows how the co-treatment (nNOSI + 6 OHDA) elicits listless/PD-like symptoms with significantly greater survival than that of single treatments of either 6 OHDA or nNOSI (* *p* < 0.0001). Specifically, by 72 h of treatment only 10%–20% of either 6 OHDA and nNOSI fish survived their respective treatments. However, approximately 90% of co-treated fish survived (** *p* < 0.0001) during the same time period; (**B**) the effect of co-treatment duration periods on washout recovery from the listless phenotype. The fish that were exposed to the co-treatment for 24 h then washed out the next day (Co-T 24) had all returned to a normal state after being nearly 100% listless the day before. Fish that were washed out after being in the same co-treatment solution for 48 h (Co-T 48) spiked at the 48 h mark (approximately 90% listless) then after washout steadily recovered over the next 48 h but more slowly than the Co-T 24 fish reaching only a 40% recovery rate. The samples that were washed out after 72 h of co-treatment (Co-T 72) were 100% listless by the second day and stayed there until wash out on day 3. However, only approximately 10% recovered to their normal state at 24 h post washout. In conclusion, Co-T 24 fish completed recovered from ERS washout, In contrast, Co-T 48 fish recovered more slowly than the Co-T 24 group (* *p* < 0.001) but faster than the co-T 72 h washout fish (** *p* < 0.001). Arrows indicate time of co-treatment washout. Bars equal ± SD.

Figure 4 shows an analysis of the ability of 6 dpf treated fish to recover from 6 OHDA, nNOSI, and nNOSI + 6 OHDA co-treatments under various conditions. Figure 4A shows how the co-treatment (nNOSI + 6 OHDA) elicits listless symptoms with significantly greater survival than that of single treatments of either 6 OHDA or nNOSI (*p* < 0.0001). Specifically, by 72 h of treatment, only 10%–20% of either 6 OHDA and nNOSI fish survived their respective treatments. However, a significantly higher percent (approximately 90%) of co-treated fish survived (*p* < 0.0001) during the same time period. Figure 4B shows the effect of co-treatment duration periods on washout recovery from the listless phenotype. Specifically, fish that were exposed to the co-treatment for 24 h then washed out the next day (Co-T 24) had all returned to a normal state after being nearly 100% listless the day before. Fish that were washed out after being in the same co-treatment solution for 48 h (Co-T 48) spiked at the 48-h mark (approximately 90% listless), then, after washout, steadily recovered over the next 48 h, but more slowly than the Co-T 24 fish reaching only a 40% recovery rate. The group that was washed out after 72 h of co-treatment (Co-T 72) was 100% listless by the second day and stayed there until washout on day 3. However, only approximately 10% were able to recover to their normal state at 24 h post washout. In conclusion, Co-T 24 fish completely recovered from ERS washout, In contrast, Co-T 48 fish recovered more slowly than the Co-T 24 group (*p* < 0.001) but faster than the co-T 72 washout fish (*p* < 0.001). Arrows indicate time of co-treatment washout.

### 3.5. l-DOPA Treatment Restores Sensory-Motor Deficits Initiated in NO Deficient Fish

**Figure 5:**
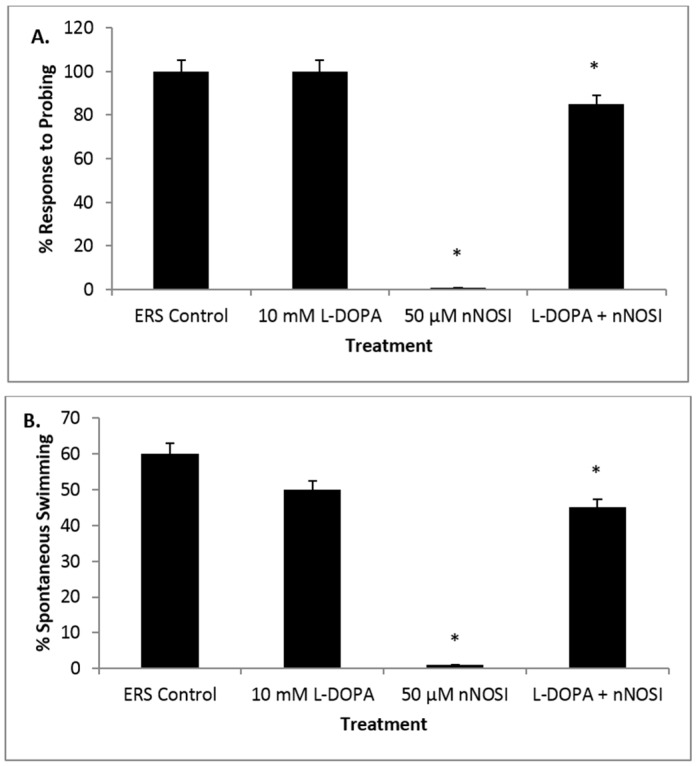

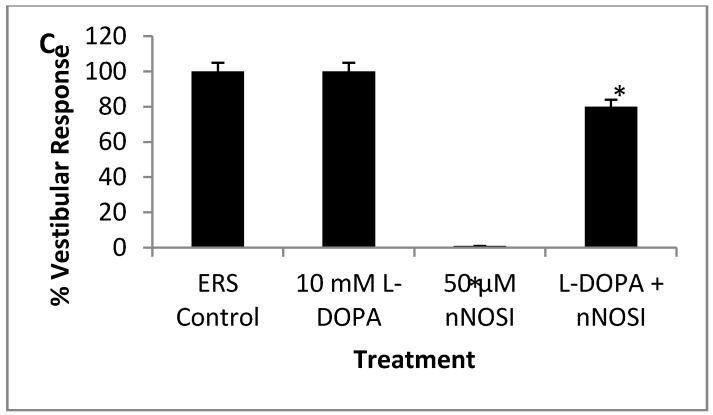
Graphs representing the three components of the listless condition and their appearance in the fish population under various l-DOPA and nNOSI treatment conditions. (**A**) This figure demonstrates the effect of treatment on percentage of zebrafish that respond to probing. After 48 h of treatment, 85% of fish co-treated with both l-DOPA and nNOSI responded to probing compared to 0% of fish treated with only nNOSI (* *p* < 0.0001). In addition, 100% of fish in both the control and l-DOPA groups responded to probing. Additionally, note that there is no significant difference among the ERS control, l-DOPA, and co-treated groups (* *p* > 0.05); (**B**) this figure shows the effect of treatment on percentage of zebrafish that swim spontaneously without being probed. One hundred percent of fish from all groups survived. After 48 h of treatment, 45% of fish co-treated with both l-DOPA and nNOSI swam spontaneously without being probed, compared to 0% of fish treated with only nNOSI (* *p* < 0.001). In addition, 60% of control fish and 50% of l-DOPA treated fish were observed spontaneously swimming. Additionally, note that there is no significant difference among the ERS control, l-DOPA, and co-treated groups (* *p* > 0.05); (**C**) this figure demonstrates the effect of treatment on percentage of zebrafish that displayed a vestibular (righting) response. After 48 h of treatment, 80% of fish co-treated with l-DOPA and nNOSI were able to maintain their balance, compared to 0% of fish treated with only nNOSI (* *p* < 0.0001). In addition, 100% of fish in both the control and l-DOPA groups were able to maintain their balance. Additionally, note that there is no significant difference among the ERS control, l-DOPA, and co-treated groups (* *p* > 0.05). Bars indicate ± SD. In addition, note that 100% of fish from all groups survived.

Figure 5 graphs represent the three components of the listless condition and their appearance in the fish population under various treatment conditions. Figure 5A demonstrates the effect of treatment on percentage of zebrafish that respond to probing. After 48 h of treatment, 85% of fish co-treated with both L-DOPA and nNOSI responded to probing compared to 0% of fish treated with only nNOSI (*p* < 0.0001). Figure 5A also shows that 100% of fish in both the control and l-DOPA groups responded to probing. Additionally, note that there is no significant difference among the ERS control, l-DOPA, and co-treated groups (*p* > 0.05). Figure 5B shows the effect of treatment on the percentage of zebrafish that swim spontaneously without being probed. After 48 h of treatment, 45% of fish co-treated with both l-DOPA and nNOSI swam spontaneously without being probed, compared to 0% of fish treated with only nNOSI (*p* < 0.001). In addition, 60% of control fish and 50% of l-DOPA treated fish were observed spontaneously swimming. Additionally, note that there is no significant difference among the ERS control, l-DOPA, and co-treated groups (*p* > 0.05). Figure 5C demonstrates the effect of treatment on percentage of zebrafish that displayed a vestibular (righting) response. After 48 h of treatment, 80% of fish co-treated with l-DOPA and nNOSI were able to maintain their balance, compared to 0% of fish treated with only nNOSI (*p* < 0.0001). In contrast, 100% of fish in both the control and l-DOPA groups were able to maintain their balance. Additionally, note that there is no significant difference among the ERS control, l-DOPA, and co-treated groups (*p* > 0.05). In addition, 100% of fish from all groups survived (data not included).

## 4. Discussion

The basal ganglia (BG) are a collection of subcortical grey matter nuclei that is, among other functions, responsible for the modulation of motor activity. There are a number of neurological conditions associated with BG dysfunction, such as Parkinson’s disease (PD). Various neurotransmitters serve to regulate these motor functions in the BG, one of which is dopamine (DA). It is now well established that DA neurons located in the substantia nigra compacta (SNc) innervate, not only the striatum, but also other basal ganglia nuclei through what is called the nigrostriatal pathway. Damage to the nigrostriatal pathway and the degeneration of dopaminergic neurons in the SNc causes motor dysfunctions associated with PD.

Several signaling molecules have been implicated in the neuromodulation/neuroprotection of DA neurons in the nigorstrital (BG) pathway. One of these signaling molecules is NO. By virtue of its gaseous state, NO can diffuse across cellular membranes without the aid of membrane bound transport proteins or receptors and can interact directly with its end targets either in the cell in which it was synthesized or in surrounding cells. In turn, its actions are precisely controlled due to its very short half-life and restricted diffusion distance [8,9]. Specifically, in the BG, NO has been shown to affect DA release, influence transporter function, and elicit neuroprotection of DA neurons [18]. Specifically, of the four NOS isoforms, nNOS is the predominant source of NO in neurons of the BG and has been implicated in movement disorders like PD [34]. NO synthesis is regulated in the striatum of the BG by both the neurotransmitters glutamate and DA [18]. NO synthesis has been shown to take place in the medium sized spiney interneurons of the BG striatum and the major striatal projection medium-sized neurons (MSNs) possess the highest brain levels of soluble guanylyl cyclase (sGC), the major NO cellular receptor [18]. This is in agreement with the finding that, in the BG, nNOS is believed to act through the cGMP-dependent pathway, which acts to modulate transcription factors, phosphodiesterases, ion-gated channels, or cGMP-dependent protein kinases (PKG), each of which continues to act physiologically in the nervous system [17].

NO evokes the release of DA and other neurotransmitters in the striatum of the BG, as well as affects their function [18]. It has been demonstrated that at high concentrations NO can be a mediator of excitotoxic neuronal damage and binds to superoxide anion (O_2_^−^), which causes harmful pathophysiological effects [10,18]. In addition, NO can act as a free radical, which damages critical metabolic enzymes and can react with superoxide to form the even more potent peroxynitrite (OONO-) [11]. However, at lower concentrations, NO can act as a cellular protectant through the prevention of neuronal depolarization, apoptosis, and regulation of the redox state within the mitochondria [12,13]. Abnormal nNOS/NO metabolism is suggested to contribute to the pathogenesis and pathophysiology of PD [11]. Therefore, the precise regulation of NO brain levels is crucial for neurological health. E2 is another signaling/hormonal molecule linked with numerous nervous system functions including PD. Specifically, it has been well established through many in vivo and in vitro studies that E2 is critical to normal development, maintenance, and function of the central nervous system [19]. Specifically, there is a vast and complex E2 response system throughout the nervous system, including the BG, all of which have E2 receptors [19,20]. A key observation linking E2 to PD is that there is a sexual dimorphism apparent in the human population based on various clinical studies. Specifically, women generally have a lower risk for PD than men. This changes after menopause, and, therefore, E2 is implicated to be a PD protectant in the female population [20]. Part of the mechanism by which E2 may exert its influence in the BG of PD patients is through its documented influence on the expression of NOS [21]. Therefore, more research is necessary to understand the effects of the E2-NO linkage, particularly in the BG, to better understand their possible medicinal applications in PD.

BG functions, as exhibited in mammals, have been conserved throughout all species of the vertebrate world, thus attesting to its importance and utility to the regulation of motor functions [2]. Indeed, the DA system has been well characterized in both zebrafish embryo development and in adults. The DA system in zebrafish, which is equivalent to the nigrostriatal pathway in mammals, has been shown to ascend to the subpallium (striatum) from the basal diencephalon [22]. These DA neurons can be easily detected dorsally on either side of the midline between the developing eyes as early as 2–5 days post-fertilization (dpf) [23]. Their numbers, approximately 14 on either side of the midline, are large enough to visualize and quantify through techniques, such as DA, DA transporter (DAT), and vesicular monoamine transporter 2 (VMAT2) immunohistochemistry or in situ hybridization studies [23,24]. Accordingly, the hypothesis of this current study states that NO acts as a neuromodulator or neuroprotectant in the dopaminergic equivalent of the nigrostriatal pathway in the zebrafish preventing the “listless” condition of locomotor dysfunction, similar to PD-like symptoms in humans. Recent studies from our lab have begun to address this issue by establishing a zebrafish locomotor dysfunction model linked to both E2 and NO deficiency Specifically, locomotor deficient zebrafish embryos were named ‘listless’ in response to E2 deprivation caused by aromatase inhibitor (AI, inhibits the enzyme responsible for E2 synthesis) treatment [26]. At 2 days post fertilization (dph) fish, after 2–4 days of AI treatment, were unable to right themselves (vestibular) and lost their swimming capacity even when stimulated by a probe thus exhibiting sensory-motor deficits [27]. Since the literature indicated that E2 regulates NOS, we also looked at the effects of l-NAME, an inhibitor to all NOS isoforms, on fish locomotor activities and were able to elicit the same listless phenotype as with AI treatment [28]. Subsequently, it was shown that l-NAME + NO co-treatment significantly l-NAME + E2 tri-treatment prevented E2 rescue of the AI-induced ‘listless’ condition. In the present study, we substituted nNOSI for l-NAME and found similar results but more robust and elicited the listless phenotype at an order of magnitude lower concentration. Not only did these latter results imply the NO may be the downstream mediator of an E2 rescue of fish from the listless condition, but it also demonstrated that in the absence of endogenous NO fish also became listless. These data are in agreement with those nNOSI studies conducted in rodents and pigeons. Specifically, nNOSI administration to rodents was found to elicit a decrease in spontaneous motor activity and demonstrated cartalepsy [18]. In turn, treatment with either an NO precursor (l-argenine) or donor (malsidomine) attenuated the nNOSI-induced cartalepsy [18]. Only one other recent paper describes the effects of l-NAME on neurotransmitter function in zebrafish. Specifically, Maximino et al. [35] described that l-NAME pretreatment in adult fish treated with the adenosine receptor agonist IB-MECA blocked its effects on gotaxis and scototaxis. However, two other papers describe that treatment with l-NAME and DETA-NO has significant effects on the morphogenesis of zebrafish spinal cord motor neurons and neuromuscular properties [8,31]. Therefore, for the first time initial evidence is presented in the current study for E2/NO’s role in locomotor activities in an embryonic zebrafish model was established but needed further clarification regarding their role in DA-related motor functions.

Subsequently, our current study was designed to explore the involvement of both E2 and NO in response to fish treated with the neurotoxin 6-OHDA. Specifically, treatment of fish with 6-OHDA caused motor symptoms similar to the previously reported listless model, which very closely aligns with the PD behavioral phenotype in humans. For example, fish were lethargic and nonresponsive to tactile stimulation regarding swimming movements. In addition, they lacked an adequate vestibular response and were observed to lay on their sides. In turn, fish treated with 6-OHDA and either DETA-NO or E2 as a co-treatment were rescued from the listless phenotype in much the same manner as in nNOSI/co-treated fish. These findings are in agreement with studies have reported the neuropro0tective effects of both E2 and NO [12,13,19]. In addition, 6-OHDA treated fish have also been shown to affect motor behavior by partially or completely eliminating dopaminergic neurons in the DA system in zebrafish the basal diencephalon [24,25,36]. However, this study is the first report of a complete loss of motor function with 6-OHDA treatment. This could be explained due to the earlier developmental ages used previously, shorter treatment periods, and/or concentration differences. However, McKinley et al. [23] did report that MPTP treatment in 1 dpf zebrafish did cause fish to become completely lethargic and immobile 3 days after treatment and was concentration dependent. Several other studies have shown that fish can be rescued from 6-OHDA treatment by other types of co-treatments. For example, Feng et al. [37] demonstrated that both vitamin E and Sinemet (levodopa) rescued zebrafish from abnormal swimming behaviors. In conclusion, the current study reports for the first time the rescue of 6-OHDA treated fish by either l-NAME or E2 indicating their involvement in the functioning of the DA system in the zebrafish brain in eliminating the listless phenotype.

Continuing in this line of evidence for DA perturbation leading to a listless phenotype, evidence from the current study also demonstrated that NO deprived zebrafish were rescued from the listless condition when co-treated with l-DOPA, a precursor to DA used routinely in PD therapy. These results add additional evidence that NO deprivation negatively affects DA homeostasis, most likely its synthesis, release or uptake. Given that l-DOPA supplementation can correct this deficiency, it may be that NO deprivation is affecting DA synthesis or signaling, which, in turn, leads to the initiation of the ‘listless’ phenotype in fish. Indeed, past literature has indicated that NO affects the synthesis and release of DA [18]. In addition, Feng et al. [37] have shown that zebrafish treated with 6-OHDA respond positively to Sinemet (levodopa) co-treatment by partially rescuing fish from locomotor deficiencies. However, data from the current study are the first report of l-DOPA rescue of nNOSI deprived zebrafish and begins to link NO to the DA controlled locomotor functions in this model.

Evidence was also presented in the current study that suggested that response of fish to nNOSI-induced motor deficits causing the listless condition was developmentally regulated. Specifically, the optimum time to start nNOSI treatment was found to be much later in development at 5–6 dpf. Other data consistent with these findings has shown that nNOS mRNA is not fully expressed in the zebrafish brain and spinal cord until around 55 dpf [38]. This most likely explains why nNOSI treatment at 48 hpf produced sporadic and inconsistent results, which were more demonstrable by 96 hpf. Studies by Jay et al. [34] reached similar conclusions in their report of the effects of NO on the neuromuscular properties of developing zebrafish. Specifically, they concluded that since nNOS was fist expressed in the spinal cord at around 30 hpf the earliest stages of neuromuscular junction development are not likely to be NO-dependent.

Interestingly, it was also observed that the longer that fish are exposed to a 6 OHDA + nNOSI co-treatment the slower the recovery after washout, but their survival rates were significantly extended over a number of days compared to just individual treatments. Previous evidence demonstrated that 6-OHDA caused oxidative stress in dopaminergic neurons [24]. Enhanced NO levels are known to increase during tissue toxicity, resulting in cell death through their further perpetuation of oxidative stress process [10]. A logical explanation for this phenomena would be that co-treatment with nNOSI diminishes the increased presence of NO caused by 6-OHDA-induced toxicity, thus, creating a more desirable environment for cell/fish survival.

Most significantly, NO involvement in the motor homeostasis of the embryonic zebrafish was shown to be expressed through the NO-cGMP-dependent pathway. Specifically, DTT treatment had no effect on eliciting the ‘listless’ phenotype where exposure of fish to ODQ caused them all to exhibit the listless phenotype as with either NO or E2 deprivation and 6-OHDA treatment. This is in agreement with several other studies, which have determined that this pathway is the preferred one that drives the NO-mediated striatal output in mammalian models [8].

## 5. Conclusions

In conclusion, these results indicate for the first time that there is a link in embryonic zebrafish between E2, NO and DA systems that regulate motor functions. Furthermore, these results substantiate earlier findings that the zebrafish will provide an additional model for the development of further knowledge of motor disorders, such as PD and the development of new/novel therapeutic strategies [25]. Similar E2, NO, and DA relationships have recently been reported in mouse models of PD. For example, Nakaso et al. [39] describe the neuroprotective role of E2 receptors in dopaminergic neurons. In addition, Han et al. [40] reported that in an nNOS-null mutation there were the same mitochondrial electron transport chain (ETC) enzyme deficits as seen in the PINK1-null mutation. In turn, optimum levels of NO enabled PINK1-null dopaminergic neuronal cells to regain the mitochondrial translocation of Parkin, which appeared significantly suppressed by the nNOS-null mutation. This suggests the feasibility of NO-based pharmacotherapy for defective mitophagy and ETC enzyme deficits in PD.
